# American Medical College Association

**Published:** 1877-07

**Authors:** 


### Editorial.

### AMERICAN MEDICAL COLLEGE ASSOCIATION.

In our last issue we had only sufficient space to give the general proceedings at the recent meeting of this Association. The Constitution, By-Laws, and Articles of Confederation, adopted at the permanent organization in Chicago, will be found below. It will be observed that no very decided innovation was made on the established usages of colleges.

The unqualified condemnation of two graduating courses a year by the same Faculty, only expresses the sentiments previously entertained by a very large majority of medical colleges in this country.

Most institutions had already increased the time for a course of lectures nearly to that adopted by the Association. Seventeen weeks, the minimum time understood universally to govern medical colleges, has been extended to nineteen by some, and even to twenty-one by some others. So that twenty weeks required by the "Articles of Confederation," amount to no very decided change in this particular.

The knotty question of fees has been passed over in a manner not to come directly in conflict with any. "Established fees" are alluded to without any expression as to the amount. The difficulty heretofore mentioned in this JOURNAL has been avoided. Entire uniformity in amount being impracticable just now, for reasons we have already given, the Association wisely ignored the question.

Cash payment of fees, and the limited number of beneficiaries, outside of those required by the charter of certain colleges, will not, we imagine, be particularly objectionable to the faculties of the various medical colleges throughout the land.

CONSTITUTION.

ARTICLE I.—*Name.*—The name of this Association shall be the "American Medical College Association."

ARTICLE II.—*Objects.*—The objects of this Association shall be the advancement of medical education in the United States, and the establishment of a common policy among medical colleges in the more important matters of college management.

ARTICLE III.—*Officers.*—The officers shall be a president, a vice-president, and a secretary and treasurer, who shall severally perform the usual duties of such officers. They shall be elected by ballot at each session of the Association, and shall serve from the time of adjournment of such session till the adjournment of the next ensuing session; but present officers shall be perpetually eligible to re-election.

ARTICLE IV.—*Membership.*—Section 1. Any regular chartered medical school in the United States which shall conform and subscribe to the Articles of Confederation of this Association may become a member of the same.

Sec. 2. Any college member which shall be convicted, upon trial, of any willful violation of any of the Articles of Confederation of the Association, or which shall fail to make answer to such charges of such violation, duly preferred, at the meeting at which the same come up for trial, shall thereby forfeit its membership in the Association.

Sec. 3. Any college which shall thus have forfeited its membership, or any college whose original application for membership shall have been rejected by the Association, shall not be eligible for membership again until a period of two years shall have elapsed since the time of forfeiture or rejection.

ARTICLE V.—*Meetings.*—Meetings of the Association shall be held not oftener than annually, at some time in the months of May or June. The exact time and place shall be fixed by the President with regard to the object for which the meeting is to be held; but when, in his judgment, there is no special reason to the contrary, preference shall be given to the place of meeting of the American Medical Associan, and to a time conveniently near to the time of meeting of the same.

Article VI.—*Representation and Voting.*—At the meeting of the Association each college may be represented by one or more delegates, or may submit, in writing, resolutions, amendments to the Constitution or By-laws, or its vote upon pending questions, except upon charges against a college for violating the Articles of Confederation. In all cases each college shall have but a single vote; and when representation is by two or more delegates, the vote shall be cast by the senior delegate in the faculty rank. The president shall not be deprived by his office of his privileges as delegate in voting. Upon questions connected with the trial of charges against a college, the accusing and accused college shall have no vote.

Article VII.—*Amendments.*—Amendments to the Constitution may be proposed at the meetings of the Association or by letter to the Secretary. In order to be entertained, they must be seconded, at the time of proposal, by one other college or its delegate. When proposed at a meeting, or submitted to the Secretary after call for a meeting has been issued, they must lie over a year before a vote is taken. Upon voting, two-thirds of the votes cast shall be necessary for adoption.

BY-LAWS.

Article I.—*Calls for Meetings.*—The calls for meetings shall be issued by the Secretary, after conference with the President as to time and place, and must not be later than two months before the proposed time of meeting. The secretary shall call meetings upon:

1. Direction of the president, who is empowered to convene the Association at his pleasure.

2. Resolution of adjournment at previous meeting.

3. Receipt, before March 1, of proposed amendments to the Constitution, By-laws, or Articles of Confederation, duly recommended by two colleges, or of a written request for a meeting for any purpose, when proffered by not less than three colleges.

4. Receipt, before January 1, of charges and specifications against a college for violation of the Articles of Confederation.

In all cases, where a call for a meeting is issued, the object of the same must be stated in the call, with proposed amendments, resolutions, or charges given in full.

Article II.—*Order of Business at Meetings.*—The order of business at meetings shall be as follows:

1. Presentation of credentials of delegates.
2. Reading of minutes of last meeting.
3. Voting upon pending questions.
4. Reading of annual reports of members.
5. Introduction of new business.
6. Election of officers for the ensuing term.
7. Treasury report of the Secretary.
8. Adjournment.

Article III.—*Admission to Membership.*—Section 1. Colleges which shall, through their delegates, subscribe to the Constitution, By-laws, and Articles of Confederation, at the meeting of the Provisional Association of Medical Colleges, at which the same shall have been adopted, and shall file with the Secretary a copy of their college charter, shall thereby become members of the Association.

Sec. 2. After adjournment of the aforesaid meeting, colleges wishing to join the Association must apply to the Secretary, and forward a copy of their college charter. The Secretary shall thereupon send notice of such application to all the colleges of the Association. If, after four months, no objection shall have been received, the Secretary shall then send to the applicant a copy of the Constitution, By-laws, and Articles of Confederation, and, on return of the same, duly subscribed by an authorized officer of the college, shall enter the name of said college upon the roll of members of the Association. Objection to the application on the ground of non-conformity to the Articles of Confederation may be made by any college member; but the same must be submitted in writing to the Secretary, and within four months after receipt of the notice of application. Such objection must be in the form of definite charges and specifications. Upon the receipt of a written objection, the Secretary shall immediately forward the same to the applying college; and, where the objection shall have been received before January 1, shall call a meeting of the Association, for the ensuing season, to consider the same. At this meeting, the applying college shall be allowed to send a member of its faculty, to conduct its case before the Association. The consideration of the objections shall then be conducted in the manner prescribed in Article IV, for the trial of charges against a member, and a two-thirds vote shall be necessary for the admission of the applicant.

Sec. 3. Any college which shall have been disqualified for membership for two years, as provided in Article IV, Section 3, of the Constitution, and which shall, at the expiration of that period, desire to join the Association, must make application again in the regular form.

ARTICLE IV.—*Charges Against Members.*—Section 1. Charges against a college member for violation of any of the Articles of Confederation, shall be entertained only when preferred by a college member of the Association, and occompanied by definite specifications. Such charges and specifications must be submitted in writing, to the Secretary, on or before January 1. Upon receipt the Secretary shall immediately transmit the same to the accused college, and, unless the charges be, in the meantime, withdrawn, shall convene the Association for the ensuing season for trial of the accusation.

Sec. 2. At the trial of the charges against a member, the accusing college must be represented by delegate; but the accused may, at its option, submit a written defence only, without representation by delegate. The case for the prosecution shall first be submitted, and then that for the defence. No counsel shall be allowed, but the witnesses on either side may be cross-examined by any delegate present. The Association shall decide, by majority vote, upon the admissibility of evidence, or of questions to witnesses. Upon closure of the case, a vote shall be taken thereon, before the final adjournment of the session, and a two-thirds vote shall be necessary for conviction.

Sec. 3. In case of failure by an accused college to make answer to the charge at the meeting of the Association duly convened for trial of the same, the meeting may, by majority vote, drop the accused college from the roll of members; and such dropping shall be equivalent to forfeiture of membership for conviction of violation of the Articles of Confederation.

Sec. 4. No member shall be tried twice upon the same specifications.

ARTICLE V.—*Delegates.*—Section 1. Delegates to the meetings of the Association must be chosen from among the executive officers of a college, or from the members of the faculty having a vote on the passage of candidates for graduation.

Sec. 2. In case objection is made to any delegate upon grounds of personal unfitness, the case shall be argued before the meeting, and by a two-thirds vote the delegate may be excluded. But, in such case, the retiring delegate shall be entitled to file with the Secretary such communications or votes upon pending questions (except upon charges against a member or applicant) as he may have been instructed by his college to present to the meeting.

ARTICLE VI.—*Affiliated Colleges.*—Section 1. The list of American affiliated colleges to be recognized by the Association shall include only the following: 1. Members of the Association; 2. Colleges not members, which are not ineligible for membership, and which conform to the following of the Articles of Confederation: Article I, sections 1 and 3 of Article II, Article III, Article V.

Sec. 2. The Secretary shall send a copy of this Article and of the Articles of Confederation to all American medical colleges not members of the Association, with a request to know if they desire to be placed upon the affiliated list, and if they conform to the requirements demanded. Upon receipt of an affirmative answer, accompanied by a copy of the college charter, he shall notify all members of the Association of the fact, and the admission of the college to the affiliated list shall then be proceeded with in the same manner as provided in Article III, for admission to membership.

Sec. 3. Charges against an affiliated college shall be proceeded with in the same manner as provided in Article IV, for proceedings in case of charges against a member.

Sec. 4. Upon conviction of non-conformity with the necessary requirements for a place upon the list of affiliated colleges, the convicted college shall be dropped from the same, and shall not be eligible for re-establishment till a period of two years shall have elapsed.

ARTICLE VII.—*Reports.*—Section 1. Every college member and every affiliated college shall forward, yearly, to the secretary of the Association as many copies as there are colleges in the Association, of every catalogue, circular, announcement, prospectus, form of advertisement, or other publication issued by the college during the year, and shall also make a full and true report embracing the following points: 1st. Honorary degree conferred within the year, with the name and age of the recipient, and the reasons why the degree was given; 2d. The names of all persons who have been allowed remissions or reductions of established fees, with the reasons, in each case, for the proceeding. If no honorary degrees have been conferred or reductions or remissions of fees made, the fact shall be reported.

Sec. 2. At each meeting of the Association the printed documents specified in section 1 shall be distributed among the delegates present, and the special reports as to honorary degrees and reductions and remissions of fees shall be read to the Association by the Secretary.

Sec. 3. The documents and reports called for in section 1 must be forwarded to the Secretary within four weeks after the annual commencement of the college. In case of failure to do so on the part of any college, the Secretary shall notify the delinquent af its failure, and if the reports be not received within four weeks after issuing such notice, the delinquent college shall be suspended in its membership of the Association, and in its privileges as an affiliated college, until the required reports be made. In such case, the Secretary shall notify both the delinquent and all the colleges of the Association of the suspension, and, upon the receipt of the reports, of the reinstatement of the delinquent college.

Article VIII. *Assessments.*—Section 1. To meet expenses, the Secretary may assess the colleges of the Association in a sum not to exceed $10 a year.

Sec. 2. He shall account for his receipts and expenditures at each meeting of the Association.

Sec. 3. Any college which shall be two years in arrears shall be notified of the fact by the Secretary, and if the dues be not paid within three months thereafter the delinquent college shall be suspended in its right of representation in the Association until payment be made.

Article IX.—*Amendments.*—Medical colleges belonging to this Association shall publish the Articles of Confederation in their annual catalogues for at least three consecutive years after joining the Association.

ARTICLE X.—Amendments to these By-laws shall be proposed and adopted in the manner prescribed for amendments to the Constitution.

ARTICLES OF CONFEDERATION.

(*To be subscribed and conformed to by all the Colleges of the Association.*)

ARTICLE I.—*Of the Faculty.*—The medical members of the faculty must be *regular* graduates or licentiates and practitioners of medicine, in good standing, using the word "regular" in the sense commonly understood in the medical profession.

ARTICLE II.—*Of Tuition.*—SEC. 1. The scheme of tuition shall provide for a yearly systematic course of instruction covering the general topics of Anatomy, including dissections, Physiology, Chemistry, Materia Medica and Therapeutics, Obstetrics, Surgery, Pathology, and Practice of Medicine. The collegiate session, wherein this course is given, shall be understood as the "regular" session.

SEC. 2. Said regular session shall not be less than twenty weeks in duration. This section to go in force at and after the session of 1879–80.

SEC. 3. Not more than one regular session, counting the regular session as one of the two courses of instruction required for graduation, shall be held in the same year.

ARTICLE III.—*Requirements for Graduation.*—No person, whether a graduate in medicine or not, shall be given a diploma of "Doctor of Medicine" who shall not have fulfilled the following requirements, *except* as hereinafter provided for in Article IV:

1. He must produce satisfactory evidence of good moral character, and of having attained the age of twenty-one years.

2. He must file a satisfactory certificate of having studied medicine for at least three years under a *regular* graduate, or licentiate and practitioner of medicine, in good standing, using the word "regular" in the sense commonly understood in the medical profession. No candidate shall be eligible for final examination for graduation unless his term of three years' study shall have been completed, or shall expire at a date not later than three months after the close of the final examination. This section to take effect at and after the session of 1879-80.

3. He must file the proper official evidence that, during the above-mentioned three years, he has matriculated at some affiliated college, or colleges, for two regular sessions, and in the course of the same (except as provided in 4,) has attened two full courses of instruction on the seven topics mentioned in Article II. But the *latter*, at least, of the two full courses must have been attended at the college issuing the diploma. No two consecutive courses of instruction shall be held as satisfying the above requirements unless the time between the beginning of the first course and the end of the second is greater than fifteen months.

4. In case a college shall adopt a systematic graduated scheme of tuition, attendance on the whole of the same shall be equivalent to the requirements mentioned in 3, *provided* such scheme includes instruction in the seven topics mentioned in Article II, and requires attendance at least at two yearly regular collegiate sessions of not less than twenty weeks' duration each.

5. The candidate must have passed a personal examination before the faculty on all seven of the branches of medicine mentioned in Article II.

6. He must have paid in full all college dues, including the graduation fee.

Article IV.—*Of Honorary Degrees.*—An honorary degree of "Doctor in Medicine" may be granted, in numbers not exceeding one yearly, to distinguished physicians or scientific men of over forty years of age. But in such case the diploma shall bear across the face the words "Honorary" in conspicuous characters, and the same word shall always be appended to the name of the recipient in all lists of graduates.

Article V.—*Of Fees.*—Sec. 1. All fees shall be paid in lawful money, and no promissory notes or promises to pay shall be accepted in lieu of cash for payment of fees.

Sec. 2. No ticket or other certificate of attendance upon college exercises shall be issued to any student until the dues for the same shall have been fully paid.

Sec. 3. The established fees for the exercises of the regular session—except the matriculation fee, graduation fee, fee for dissections—may be reduced not more than one half to graduates of other affiliated colleges of less than three years' standing, and to undergraduates of the same who have already attended two full courses of instruction of the regular session.

Sec. 4. The same fees may be remitted altogether to a college's own alumni, to graduates of other affiliated colleges of three year's standing (the three years dating from the time of graduation, and ending at the close of the regular session for which the tickets are given,) to undergraduates who have already attended two full courses of the instructiou in the regular session, the latter of which at least shall have been in the college making the remission, and to theological students, when not candidates for a diploma.

Sec. 5. The same fees may be reduced or remitted to deserving, indigent students, to a number not exceeding *five* per cent. of the number of matriculates at the previous regular session of the college.

Sec. 6. Under no circumstances whatever other than the above shall the faculties, or any members of the same, grant *upon their own authority* any remissions or reductions of established fees. And it is distinctly understood and agreed that the faculties will discountenance and oppose the authorizing by governing boards of the admission of individual students on other than the regularly established charges for their grade.

Sec. 7. Remissiou or reduction of fees for other exercises than those of the regular session, return to a student of any moneys after payment of fees, or an appropriation of funds of the college for payment of any student's fees, or part thereof, shall be deemed violation of provisions of this article in regard to remission or reduction of fees.

Article VI.—*Of Recognition of other Colleges.*—No college shall admit to the privileges accorded in Articles III and V the students or graduates of any college which, during any period of the student's or graduate's pupilage, shall have been excluded from the list of affiliated colleges recognized by the Association.

Article VII.—*Amendments.*—Amendments to these articles shall be proposed and adopted in the manner prescribed for amendments to the Constitution.

